# Estimation of serum iron, serum lipids and serum liver enzymes in celiac disease patients of Saudi Arabia

**DOI:** 10.12669/pjms.38.8.6237

**Published:** 2022

**Authors:** Muhammad Ikram Ullah, Abdullah Alsrhani, Muhammad Atif, Irfan Shaukat, Shahid Hussain, Hasan Ejaz

**Affiliations:** 1 Muhammad Ikram Ullah, PhD, Department of Clinical Laboratory Sciences, College of Applied Medical Sciences, Jouf University, Al Jouf 72388, Saudi Arabia; 2 Abdullah Alsrhani, PhD, Department of Clinical Laboratory Sciences, College of Applied Medical Sciences, Jouf University, Al Jouf 72388, Saudi Arabia; 3 Muhammad Atif, M.Phil, Department of Clinical Laboratory Sciences, College of Applied Medical Sciences, Jouf University, Al Jouf 72388, Saudi Arabia; 4 Irfan Shaukat, PhD. Department of Biochemistry University of Narowal, Pakistan; 5 Shahid Hussain, M.Sc. Department of Pathology & Lab Medicine, Immunopathology Unit, College of Medicine, King Khalid University Hospital, King Saud University Riyadh-11451, Saudi Arabia; 6 Hasan Ejaz, PhD, Department of Clinical Laboratory Sciences, College of Applied Medical Sciences, Jouf University, Al Jouf 72388, Saudi Arabia

**Keywords:** Celiac disease, Human tissue transglutaminase IgA, Serum iron, Serum lipids, Liver enzymes

## Abstract

**Objectives:**

To evaluate the serum biochemical levels in celiac disease (CD) patients.

**Methods:**

It was a cross-sectional study carried out on 70 subjects, including 40 patients with CD and 30 healthy controls. This study was conducted at Jouf University from November, 2020 to October, 2021. The collected blood specimens were used to perform serum iron, serum lipids, liver enzymes, and human tissue transglutaminase IgA antibodies (anti-HTTG). The hematological parameters including hematocrit and MCV were determined to establish the diagnosis of iron deficiency.

**Results:**

Serum iron was significantly lower in patients as compared to the controls. Serum iron, serum HDL, blood hematocrit and MCV were significantly lower in patients than in controls (p = 0.000). Serum levels of liver enzymes (ALT and AST) and serum human tissue transglutaminase antibodies (anti-HTTG) were significantly higher in patients than in controls (p = 0.000). The correlation studies established the negative correlation of anti-HTTG IgA with serum iron (r = -0.991, p = 0.000), hematocrit (r = -0.967, p = 0.000) and MCV (r = -0.946, p = 0.000) in patients.

**Conclusion:**

The serum iron was remarkably reduced in CD patients. A negative correlation was found between anti-HTTG IgA and serum iron, while a positive serum iron was correlated with hematocrit and MCV in CD patients.

## INTRODUCTION

Celiac disease (CD) is a complex metabolic and autoimmune disorder associated with the malabsorption of gluten. Gluten protein is mainly found in wheat, rye, barley and is absorbed in the small intestine. Glutenin and gliadin proteins form a complex that leads to immune activation in patients.^1^ These immune reactions primarily affect the small intestine (mainly the proximal part), leading to the malabsorption and deficiency of minerals and vitamins.^2^ The clinical presentations of CD are widely heterogeneous^3^ and are misdiagnosed as overlapping bowel disease and other diseases.^4^,^5^ The most specific, sensitive, and preferred test is the human tissue transglutaminase (HTTG) IgA antibody, which may establish the CD diagnosis alone.^6^,^7^ The pathogenetic mechanism of celiac disease is not completely known, although it might be due to the combination of genetic, environmental, and immunological factors.^8^,^9^

It is estimated that 1% of CD cases occur in America, and the cases have been reported globally. The CD prevalence has been reported to increase gradually with improved diagnostic practices.^6^ Hence, the prevalence of CD is variable from 0.33% to 2.5% globally. In Western Sahara, the highest prevalence was found, up to 5.6%.^10^ The occurrence of CD in Saudi Arabia ranges from 0.5% and 1%.^11^,^12^

The proximal small intestine has a role in mineral absorption like iron, but chronic inflammation in celiac disease affects the absorptive capacity of the proximal small intestine. The mucosal damage leads to the development of iron deficiency due to loss of iron absorption site.^1^,^13^,^14^ The entry of iron in duodenal mucosa requires a divalent metal transporter (DMT1) which helps iron absorption parallel to the DMT1 expression.^15^ Iron has an essential role in the heme group of hemoglobin for the transport of oxygen. Iron is also crucial for cytochromes and other proteins like myoglobin.^1^

Moreover, CD may affect the liver due to the hallmark injury of the small intestine. The presentations of liver dysfunction are nonspecific types of hepatitis, such as discontent and exhaustion.^16^ CD-associated liver disease is probably developed by variable factors, comprising intestinal bacterial overgrowth, chronic infection and inflammation, gut permeability, genetic, molecular mimicry, and predisposition of genes. Liver diseases may develop as primary biliary cirrhosis, autoimmune hepatitis, and non-alcoholic steatohepatitis. These liver defects can be improved by the intervention of a gluten-free diet (GFD), particularly when morbid obesity is not present.^17^

There are various studies in which the role of lipids has been reported. Mostly, hypocholesterolemia is associated with celiac disease, which is more severe in men than in women.^18^ The possible mechanism of this understanding is due to the loss of mucosa of the small intestine that cannot absorb the cholesterol in blood circulation. Our study aimed to evaluate the role of biochemical profiles (serum iron level, lipids level, and liver enzymes) and also to determine blood constituents (hematocrit, mean cell volume (MCV) in patients with celiac disease (CD) in the Saudi population.

## METHODS

Prior to starting the study, ethical permission was obtained from the Local Ethical Board of Jouf University (HAP-13-s-001), and Helsinki Declaration (modified 2013) was followed to include the subjects. In this cross-sectional study, a total of 70 subjects comprised of 40 celiac disease patients and 30 healthy controls (sample size calculation was carried out by applying a statistical formula keeping power of study 5% and frequency of disease in the population 0.5%) were investigated. A random selection technique was applied to collect samples. The experiments were carried out at the Department of Clinical Laboratory Sciences from November 2020 to October 2021. The celiac disease patients were included with features; a) clinical features like diarrhea and weight loss; b) endoscopic and pathological features like duodenal atrophy and goblet cell hyperplasia on small intestine biopsy; and c) biochemical testing of antibodies (positive human tissue transglutaminase (HTTG) IgA antibody. The patients were screened based on the positive biopsy and high antibody levels. The patients with malabsorption syndrome, dermatitis herpetiformis, Wilson’s disease, and Crohn’s disease were excluded from the study.

After written informed consent, about five milliliters of blood was collected aseptically into serum separating gel and EDTA vacutainers for biochemical (serum iron, lipid profile and liver enzymes) and hematological analyses, respectively. Sample analyses for hematological and biochemical were carried out according to the standard protocols.

The concentration of hematocrit (Hct) and mean cell volume (MCV) was recorded in an auto-analyzer for cell counting (Sysmex 21). Serum biochemical levels of iron (Fe), serum liver enzymes (AST, ALT, and ALP), and lipids profile were analyzed by an automated chemistry analyzer (Dimension Vista 1500 Analyzer, Siemens). Determination of human tissue transglutaminase (HTTG) IgA antibodies was done by ELISA technique that detects semi-quantitatively IgA autoantibodies to tissue transglutaminase (TTG) protein in human serum (ETI-MAX3000, Diasurin).

Statistical analysis of the data was done using SPSS v.26.0. The normally distributed quantitative variables were given as mean and standard deviation (SD) and non-normally distributed as the median and interquartile range (IQR). The comparison of groups for normally distributed data was analyzed by the Student “t” test and Mann-Whitney U test. Association was carried out by Spearman’s rho correlation (rho) and Pearson correlation (r). The categorical data were compared between studied groups by Chi-square test. P-value <0.05 was considered statistically significant.

## RESULTS

This study was comprised of 70 individuals, including 40 cases of celiac disease and 30 age and sex-matched healthy controls. The gender frequency of patients was 55% male (n = 22) and 45% females (n= 18). On the other hand, the gender frequency of controls was 46.7% (n = 13) males and 53.3% females (n = 17).

The mean ages of celiac disease patients and healthy individuals were 30.9 ± 6.8 (mean ± SD) and 31.5 ± 9.0 years, respectively, without any statistically significant difference (p = 0.774). The comparison of serum iron (Fe), serum high-density lipoprotein (HDL) and blood hematocrit and means cell volume (MCV), which were significantly reduced in CD patients in comparison to healthy controls (p <0.05) is shown in Table-I. The serum liver aminotransferases (ALT and AST) and serum anti-HTTG IgA antibodies were significantly high in CD patients as compared to the controls (p <0.05).

**Table-I:** Comparison of biochemical and hematological parameters between CD patients and controls (n=70).

Parameters	Patients (mean ± SD) n = 40	Controls (mean ± SD) n = 30	Mann-Whitney U Test*/ t -test^†^	p-value
Age (years)	30.9 ± 6.8	31.5 ± 9.0	24.7*	0.774*
Iron level (11 - 31 µmol/L)	8.5 ± 4.2	18.9 ± 5.9	13.0^†^	0.000^†^
AST (10-35 U/L)	76.9 ± 9.2	36.0 ± 13.8	4.39*	0.000*
ALT (5-40 U/L)	66.8 ± 8.8	30.7 ± 10.1	31.4^†^	0.000^†^
ALP (40-125 U/L)	82.8 ± 11.4	77.7 ± 15.1	1.176*	0.135*
TG (<150 mg/dL)	100.2 ± 13.4	106.9 ± 21.3	1.184*	0.136*
T. cholesterol (<200 mg/dL)	154.2 ± 17.9	152.9 ± 11.6	1.70*	0.722*
HDL (>45 mg/dL)	49.8 ± 5.6	53.6 ± 6.4	3.87*	0.011*
LDL (<130 mg/dL)	72.3 ± 17.1	72.3 ± 11.5	2.47*	0.963*
Hct (38-48%)	28.0 ± 4.1	38.8 ± 6.0	4.248*	0.000*
MCV (80-97fl)	72.8 ± 6.0	84.5 ± 6.5	11.7^†^	0.000^†^
HTTG-IGA (U/mL)	217.4 ± 58.4	18.5 ± 9.5	4.248^†^	0.000^†^

* p-value generated by Mann-Whitney U Test;

† p-value generated by Independent Sample "t"-Test; p-value ≤ 0.05 is considered statistically significant.

The correlation between anti-HTTG IgA, serum Fe, MCV, and hematocrit was analyzed in groups. A strong negative correlation was found between anti-HTTG-IgA and serum Fe levels with r-value -0.991 (p = 0.000) in the patients’ group. On the other hand, serum anti-HTTG-IgA was negatively associated with hematocrit and MCV in the patients’ group (Table-II). A weak negative association between anti-HTTG-IgA and serum Fe levels with an r value -0.401 (p = 0.028) was also observed in apparently healthy controls, while the serum anti-HTTG-IgA did not link with other parameters like hematocrit and MCV (Table-III).

**Table-II:** Correlation matrix of biochemical parameters in celiac disease patients.

Correlations (Patients)		Iron	HTTG-IgA	Hct	MCV
Iron	Pearson Correlation	1	-0.991**	0.979**	0.952**
	Sig. (2-tailed)		0.000*	0.000*	0.000*
HTTG-IgA	Pearson Correlation		1	-0.967**	-0.946**
	Sig. (2-tailed)			0.000*	0.000*
Hct	Pearson Correlation			1	0.958**
	Sig. (2-tailed)				0.000*
MCV	Pearson Correlation				1
	Sig. (2-tailed)				

** Two-tailed Pearson correlation,

* p-value ≤ 0.05 is considered statistically significant.

**Table-III:** Correlation matrix of Biochemical parameters in apparently healthy individuals.

Correlations (Controls)		Iron	HTTG-IgA	Hct	MCV
Iron	Pearson Correlation	1			
	Sig. (2-tailed)				
HTTG-IgA	Pearson Correlation	-.401*	1		
	Sig. (2-tailed)	0.028*			
Hct	Pearson Correlation	0.333	0.265	1	
	Sig. (2-tailed)	0.072	0.158		
MCV	Pearson Correlation	0.091	0.066	0.029	1
	Sig. (2-tailed)	.633	0.730	0.877	

^**^ Two-tailed Pearson correlation,

* p-value ≤ 0.05 is considered statistically significant

Association of serum iron levels, MCV, hematocrit, and anti-HTTG-IgA, were observed between patients and controls by Chi-square test, as shown in Table-IV. The categories of serum anti-HTTG IgA were divided into four groups (negative, weakly positive, positive, and strong positive), and serum iron was distributed into three categories (normal, low, and high). The CD patients were positive (100%) for anti-HTTG-IgA, which was >30 U/mL, while it was positive only in 5 (16.6%) controls. The association of higher anti-HTTG-IgA was statistically significant (*X^2^* value = 55.27, p = 0.000) and lower serum Fe was statistically significant (*X^2^* value 32.9 with p-value = 0.000) in CD patients than controls. Similarly, hematocrit and MCV were low in 98% and 82.5% of CD patients, while it was normal in most of the individuals in the control group with a statistically strong association (p <0.05).

**Table-IV:** Associations of qualitative parameters between CD patients and controls.

Variables	Categories	Patients (n = 40)0Ppp)	Controls (n = 30)	Statistics	

		Frequency (%)	Frequency (%)	X^2^	p-value
Sex	Male	22 (55)	14 (46.7)	0.47	0.48
	Female	18 (45)	16 (53.3)		
Serum Iron	Normal	10 (25)	29 (96.7)	32.9	0.000*
	Low	27 (67.5)	0		
	High	03 (7.5)	01(3.3)		
Hematocrit	Low	39 (97.5)	11 (36.7)	31.0	0.0001*
	Normal	01 (2.5)	19 (50)		
MCV	Low	33 (82.5)	09 (30)	19.68	0.000*
	Normal	07 (17.5)	21 (70)		
HTTG-IGA	1-20	----	17 (56.7)	55.27	0.000*
	21-30	------	08 (26.7)		
	31-100	01 (2.5)	04 (13.3)		
	>100	39 (97.5)	1 (3.3)		

^*^p-value ≤ 0.05 is considered statistically significant.

## DISCUSSION

In the present study, we determined the role of biochemical profiles in celiac disease patients. The impact of serum iron, lipid profile, and liver enzymes was compared. Also, the blood concentration of MCV and hematocrit were determined to assess iron deficiency anemia diagnosis. Serum iron and hematological parameters were significantly lower in CD patients than in healthy controls. The correlation study found a positive relationship between low serum with MCV and hematocrit while negatively associated with anti-HTTG-IgA antibodies.

Iron deficiency postulates the mechanism that develops malabsorption in CD patients, and iron cannot absorb into the blood. Our results are consistent with a previous study in which iron deficiency is associated with celiac disease.^19^ In another study, anemia was detected in 11% of patients^20^ and 81.5% CD patients,^14^ while low ferritin was found in 70% of cases.^20^ There are very few studies reported in the Saudi population that only targeted epidemiological studies.^12^,^13^ Micronutrient deficiencies are due to abnormal regulatory proteins having a key role in iron absorption at the stage of the enterocyte.^21^ The mechanism for iron deficiency development is not well understood.

We also determined that the serum liver enzymes, including transaminases (AST, ALT) and ALP, were significantly raised, explaining the development of liver dysfunction in celiac disease. Our results are comparable to a study conducted on the Saudi population.^22^ Although various studies documented altered or elevated serum transaminases, however, no association was found between CD patients and controls in our study. In CD cases, the development of autoimmune and cryptogenic liver defects (having a positive response with GFD) ensues.^8^ Usually, the aminotransferase elevations are mild to moderate, mainly with a low AST/ALT ratio, and show huge manifestation variability.^23^ Some other studies documented ameliorated liver functions after the adoption of GFD.^17^ The possible mechanism is likely multifactorial, and gluten-induced damage to the gut lining could lead to elevated liver aminotransferases in celiac disease.^8^

The lipid profile was also estimated, but there was no significant association except serum HDL, which was significantly low in celiac patients. The association studies were also carried out, but there was no link with CD. A previous study documented the low serum HDL-cholesterol at baseline, which was improved with GFD intervention.^24^ The mechanism of reduced HDL is likely due to intestinal inflammation or conformational changes in apo-lipoprotein. In other reports, hypocholesterolemia occurs with reduced total cholesterol, LDL, and HDL in celiac disease.^18^ The role of GFD in altered lipid profile is unclear in CD. Although some reports described the improvement in lipoprotein with saturated fat-rich food, other data reported the worsening of cardiovascular disease. In another study, raised HTTG IgA antibodies and reduced serum cholesterol were reported in CD patients compared to non-celiacs.^25^ The status of biochemical parameters is very significant in celiac disease; these biomarkers might be helpful in the diagnosis and prognosis of the disease for future prospects.

### Limitations:

The study’s population was small and limited biomarkers were used in the diagnosis.

## CONCLUSION

The serum iron was remarkably reduced in CD patients compared to the controls establishing the iron deficiency. In patients, serum levels of liver transaminases (ALT and AST) and serum anti-HTTG IgA were significantly higher. A negative correlation was found between anti-HTTG IgA and serum iron in CD patients. A positive correlation between serum iron was observed with hematocrit and MCV in CD patients. In contrast, the correlation between altered liver enzymes and lipid profiles did not observe in CD patients and controls. Moreover, establishing and exploring the roles of biochemical parameters would be helpful in disease diagnosis and tailored medicine.

### Recommendations:

***1*.** It is recommended to conduct the studies on a large sample scale to devise the mechanism for celiac disease development. ***2*.** It is proposed to utilize the results of this study to establish the differential diagnosis of celiac disease and tailored management.

### Author’s Contribution:

**MIU:** Conceived the idea, Analysis, Original draft and is responsible and accountable for the accuracy or integrity of the work.

**AA:** Methodology and analysis.

**MA:** Literature review, investigation and analysis.

**IS:** Specimen analysis and results interpretation.

**SH:** Specimen collection and literature review.

**HE:** Literature review, analysis and editing.

## References

[1] Martin-Masot R, Nestares MT, Diaz-Castro J, Lopez-Aliaga I, Alférez MJM, Moreno-Fernandez J (2019). Multifactorial etiology of anemia in celiac disease and effect of gluten-free diet:A comprehensive review. Nutrients.

[2] Deora V, Aylward N, Sokoro A, El-Matary W (2017). Serum vitamins and minerals at diagnosis and follow-up in children with celiac disease. J Pediatr Gastroenterol Nutr.

[3] Masood N, Shaikh IA (2014). Clinical presentations and biochemical profile in adult celiac disease patients in Hyderabad:Pakistan. Pak J Med Sci.

[4] Ianiro G, Bibbo S, Bruno G, Ricci R, Arena V, Gasbarrini A (2016). Prior misdiagnosis of celiac disease is common among patients referred to a tertiary care center:A prospective cohort study. Clin Transl Gastroenterol.

[5] Rashid M, Rashid H (2019). Coeliac disease in Pakistan:A bibliographic review of current research status. J Pak Med Assoc.

[6] Rubio-Tapia A, Hill ID, Kelly CP, Calderwood AH, Murray JA (2013). American College of Gastroenterology clinical guideline:diagnosis and management of celiac disease. Am J Gastroenterol.

[7] Muhammad AH, Hussain T, Masood N, Younas M, Asghar RM, Shafi MS (2016). Accuracy of anti-tissue transglutaminase IgA antibody in the diagnosis of paediatric celiac disease. J Coll Physicians Surg Pak.

[8] Kim JV, Wu GY (2021). Celiac disease and elevated liver enzymes:A review. J Clin Transl Hepatol.

[9] Aziz DA, Kahlid M, Memon F, Sadiq K (2017). Spectrum of Celiac disease in Paediatric population:Experience of Tertiary Care Center from Pakistan. Pak J Med Sci.

[10] Kang J, Kang A, Green A, Gwee K, Ho K (2013). Systematic review:worldwide variation in the frequency of coeliac disease and changes over time. Aliment Pharmacol Ther.

[11] Hvas CL, Jensen MD, Reimer MC, Riis LB, Rumessen JJ, Skovbjerg H (2015). Celiac disease:diagnosis and treatment. Dan Med J.

[12] Aljebreen AM, Almadi MA, Alhammad A, Al Faleh FZ (2013). Seroprevalence of celiac disease among healthy adolescents in Saudi Arabia. World J Gastroenterol.

[13] Saeed A, Assiri A, Assiri H, Ullah A, Rashid M (2017). Celiac disease in Saudi children:evaluation of clinical features and diagnosis. Saudi Med J.

[14] Bledsoe AC, King KS, Larson JJ, Snyder M, Absah I, Choung RS (2019). Micronutrient deficiencies are common in contemporary celiac disease despite lack of overt malabsorption symptoms. Mayo Clin Proc.

[15] Yanatori I, Kishi F (2019). DMT1 and iron transport. Free Radic Biol Med.

[16] Hoffmanova I, Sanchez D, Tuckova L, Tlaskalova-Hogenova H (2018). Celiac Disease and Liver Disorders:From Putative Pathogenesis to Clinical Implications. Nutrients.

[17] Moghaddam MA, Nejad MR, Shalmani HM, Rostami K, Mojarad EN, Aldulaimi D (2013). The effects of gluten-free diet on hypertransaminasemia in patients with celiac disease. Int J Prev Med.

[18] Stein AC, Liao C, Paski S, Polonsky T, Semrad CE, Kupfer SS (2016). Obesity and Cardiovascular Risk in Adults With Celiac Disease. J Clin Gastroenterol.

[19] Berry N, Basha J, Varma N, Varma S, Prasad KK, Vaiphei K, Dhaka N, Sinha SK, Kochhar R (2018). Anemia in celiac disease is multifactorial in etiology:A prospective study from India. JGH Open.

[20] Khatoon S, Ahmed A, Yousaf S (2018). Iron Deficiency Anaemia In Pakistan:Celiac Disease An Underlying Cause. J Ayub Med Coll Abbottabad.

[21] Montoro-Huguet MA, Santolaria-Piedrafita S, Canamares-Orbis P, García-Erce JA (2021). Iron Deficiency in Celiac Disease:Prevalence, Health Impact, and Clinical Management. Nutrients.

[22] Saadah OI, Khayat A, Abusharifah O, Alaifan MA, Kamal NM, Bin-Taleb Y (2021). Liver function changes following the introduction of a gluten-free diet in patients with celiac disease. Clin Exp Hepatol.

[23] Marciano F, Savoia M, Vajro P (2016). Celiac disease-related hepatic injury:Insights into associated conditions and underlying pathomechanisms. Dig Liver Dis.

[24] Salardi S, Maltoni G, Zucchini S, Iafusco D, Confetto S, Zanfardino A (2016). Celiac Disease Negatively Influences Lipid Profiles in Young Children With Type 1 Diabetes:Effect of the Gluten-Free Diet. Diabetes Care.

[25] Zanini B, Mazzoncini E, Lanzarotto F, Ricci C, Cesana BM, Villanacci V (2013). Impact of gluten-free diet on cardiovascular risk factors. A retrospective analysis in a large cohort of coeliac patients. Dig Liver Dis.

